# The effect of cellular nuclear function alteration on the pathogenesis of shoulder adhesive capsulitis: an immunohistochemical study on lamin A/C expression

**DOI:** 10.1186/s10195-024-00752-8

**Published:** 2024-02-21

**Authors:** Vittorio Candela, Barbara Peruzzi, Martina Leopizzi, Natale Porta, Valeria Di Maio, Benjamin Greenberg, Carlo Della Rocca, Stefano Gumina

**Affiliations:** 1 Department of Anatomical, Histological, Forensic Medicine and Orthopaedics Sciences, Sapienza University of Rome, Istituto Clinico Ortopedico Traumatologico (ICOT), Latina, Italy; 2 Multifactorial Disease and Complex Phenotype Research Area, IRCCS Bambino Gesù Children’s Hospital, Rome, Italy; 3 Dept of Medico-Surgical Science and Biothecnologies, Sapienza University of Rome, Polo Pontino, Latina, Italy

**Keywords:** Lamin A/C, Fibroblast response in adhesive capsulitis, Adhesive capsulitis etiology, Adhesive capsulitis pathogenesis, Rotator interval in shoulder adhesive capsulitis, Shoulder rotator interval

## Abstract

**Background:**

The network of intermediate filament proteins underlying the inner nuclear membrane forms the nuclear lamina. Lamins have been associated with important cellular functions: DNA replication, chromatin organization, differentiation of the cell, apoptosis and in maintenance of nuclear structure.

Little is known regarding the etiopathogenesis of adhesive capsulitis (AC); recently, a dysregulating fibrotic response starting from a subpopulation has been described within the fibroblast compartment, which suddenly turns on an activated phenotype.

Considering the key role of A-type lamins in the regulation of cellular stability and function, our aim was to compare the lamin A/C expression between patients with AC and healthy controls.

**Materials and methods:**

A case–control study was performed between January 2020 and December 2021. Tissue samples excised from the rotator interval were analysed for lamin A/C expression by immunohistochemistry. Patients with AC were arbitrarily distinguished according to the severity of shoulder flexion limitation: ≥ 90° and < 90°. Controls were represented by samples obtained by normal rotator interval excised from patients submitted to shoulder surgery. The intensity of staining was graded, and an H-score was assigned. Statistical analysis was performed (Chi-square analysis; significance was set at alpha = 0.05).

**Results:**

We enrolled 26 patients [12 male and 14 female, mean age (SD): 52.3 (6.08)] and 15 controls [6 male and 9 female, mean age (SD): 57.1 (5.3)]. The expression of lamin A/C was found to be significantly lower in the fibroblasts of patients with adhesive capsulitis when compared with controls (intensity of staining: *p*: 0.005; H-score: 0.034); no differences were found regarding the synoviocytes (*p*: > 0.05). Considering only patients with AC, lamin A/C intensity staining was found to be significantly higher in samples where acute inflammatory infiltrate was detected (*p*: 0.004).

No significant changes in levels of lamin A/C expression were documented between the mild and severe adhesive capsulitis severity groups.

**Conclusions:**

Our study demonstrated that the activity of lamin A/C in maintaining nuclear structural integrity and cell viability is decreased in patients with adhesive capsulitis. The phase of the pathogenetic process (freezing and early frozen) is the key factor for cell functionality. On the contrary, the clinical severity of adhesive capsulitis plays a marginal role in nuclear stability.

**Level of evidence:**

III.

## Introduction

Located underneath the inner nuclear membrane of the cell is a proteinaceous meshwork known as the nuclear lamina, a structure composed of a variety of proteins, including the nuclear lamins [1, 2]. Lamins are type V intermediate filaments and are structurally composed of three domains: the rod, head and tail domains [1]. Five isoforms of lamins are known, generated by alternatively splicing the LMNA gene into lamin A and C isoforms, or encoded by LMNB1 and LMNB2 genes into lamin B1 and B2/B3 isoforms, respectively [2]. Lamins have been associated with important cellular functions, including DNA replication, chromatin organization, differentiation of the cell, apoptosis and maintenance of nuclear structure, mechanical support of cells, determination of the cellular fate and regulation of genes [3]. Different pathologies, referred to as “laminopathies”, have been associated with lamin deregulation, such as cardiomyopathies, muscular dystrophy and Hutchinson–Gilford progeria syndrome (HGPS) [4, 5]. Lamins were recently found to be involved in the cell’s reactions to mechanical force [6]; in particular, an association between the alteration of lamin A/C expression and cartilaginous issues involving osteoarthritis [7] and degeneration of human intervertebral disc [8] have been proposed. Recently, a significant lower expression of lamin A/C was documented in tenocytes of patients with rotator cuff tear with respect to healthy controls [9]; in particular, a decrease in expression was correlated to increasing tear size, emphasizing the importance of early treatment since nuclear stability is maintained and the cellular function is protected by lamin A/C expression.

Adhesive capsulitis or “frozen shoulder”, the term coined by Earnest Codman, is a condition characterized by a devastating loss of shoulder motion [10]. Its prevalence has been reported to range between 2% and 5% [11–13], it mainly affects women aged 40–60 years old [11] and the correlation between aetiology and severity with hypercholesterolemia have recently been documented [14]. Pain principally involves the anterior aspect of the shoulder with extension down the arm until its distal third [15] and a positive “Coracoid pain test” [16] together with inflammation of the superior subscapularis recess on MRI [17] are common diagnostic findings. A chronic inflammation located at the shoulder’s rotator interval leads to a capsular thickening along with a process of fibrosis and capsular adherences [10]. Recently, Akbar et al. [18] demonstrated in their prospective case–control study that subpopulations within the fibroblast compartment may take on an activated phenotype, supporting the hypothesis that activated fibroblasts may be involved in regulating the inflammatory and fibrotic processes related to this disease. However, factors associated with this dysregulated fibrotic response are still unknown. Considering the key role of lamin A/C in the regulation of cellular function, our aim was to compare A-type lamins expression between patients with AC and healthy subjects. Our hypothesis was that a decreased lamin A/C expression is present in patients with AC leading to progressive cell dysfunction involved in inflammatory and fibrotic processes.

## Materials and methods

A single-centre case–control study was performed between January 2020 and December 2021. We enrolled 35 patients with AC [16 male and 19 female, mean age (SD): 50.5 (5.85)]. The diagnosis of AC was obtained after checking the active and passive range of motion, a positive coracoid pain test [14], negative X-Ray (true AP and axillary views) for shoulder osteoarthritis and MRI of the involved shoulder showing superior subscapularis recess inflammation [17]. The control group was composed of 21 patients [9 male and 12 female, mean age (SD): 56.4 (8.04)] with proximal humerus fractures submitted to surgery.

Exclusion criteria were applied: concomitant rotator cuff tear, history of recent trauma (< 3 months) on the affected shoulder (except for that responsible for the fracture in the control group), history of shoulder pain and limited shoulder range of motion before the fracture (for the control group), anti-inflammatory drugs administration during the two months before surgery, previous AC in the opposite shoulder, rheumatologic diseases, prior shoulder surgery, diabetes, hypercholesterolemia and thyroidopathies.

Patients with adhesive capsulitis were arbitrarily distinguished into two groups according to the severity of shoulder flexion limitation: group A (forward flexion ≥ 90°) and group B (forward flexion < 90°).

Cases were submitted to surgery after 3 months of failed conservative treatment. During surgery, tissue samples excised from the rotator cuff interval were analyzed for lamin A/C expression by immunohistochemistry. Normal rotator cuff interval samples, excised in patients with proximal humerus fractures with intact rotator cuff submitted to open reduction and plate fixation, were used as controls.

Immunohistochemistry was conducted on AC and control sections by following the manufacturer’s instructions with horseradish peroxidase-3, 3’-diaminobenzidine (HRP-DAB)-based kit (Dako LSAB Kit peroxidase; Dako, Carpinteria, CA). Specimens were deparaffinized and rehydrated in graded ethanol. Endogenous peroxidase activity was blocked by 3% hydrogen peroxide. Antigen retrieval was performed in EDTA buffer pH 8.0 (UCS Diagnostic). The sections were then incubated overnight at 4 °C with a rabbit anti-lamin A/C antibody (A0249, Abclonal; Woburn, MA) and subsequently stained using Dako LSAB Kit peroxidase.

The nuclear expression of A-type lamins in fibroblast and synoviocytes were evaluated by a pathologist, blinded to patient information, using two criteria: the percentage of immunoreactive cells (semiquantitative method) and the intensity of staining (qualitative method). The percentage of immunoreactive cells was estimated by a medium-magnification (20 ×) microscope examination of the entire section. The intensity of staining was graded as: negative (0), low staining (1), moderate staining (2), and high staining (3) [9]. The percentage of cells at each staining intensity level was calculated, and an H-score was assigned using the following formula: [1 × (% cells 1 +) + 2 × (% cells 2 +) + 3 × (% cells 3 +)]. The final score, ranging from 0 to 300 (H1 = 0–50; H2 = 51–100; H3 = 101–150; H4 > 150), gives more relative weight to higher intensity staining in a sample [9].

All participants signed an informed consent form in accordance with the Declaration of Helsinki and according to the law in our country this study does not need any ethics committee approval.

### Statistical analysis

All analyses were completed using GraphPad Prism 6.0. Contingency data were analysed by the Fisher’s exact test of independence. Accordingly, regarding statistical analysis requirements, the score 0 for A-type lamin intensity (negative staining) was excluded by Fisher’s exact test since no samples showed negative staining results. The level of significance was set at alpha = 0.05. SPSS version 20 (IBM, Chicago, IL) for Windows was used.

## Results

Finally, the study group was composed of 26 AC cases [12 male and 14 female, mean age (SD): 52.3 (6.08)] and 15 controls [6 male and 9 female, mean age (SD): 57.1 (5.3)], as 9 and 6 patients were excluded from the case and control groups, respectively.

**Fig. 1:**
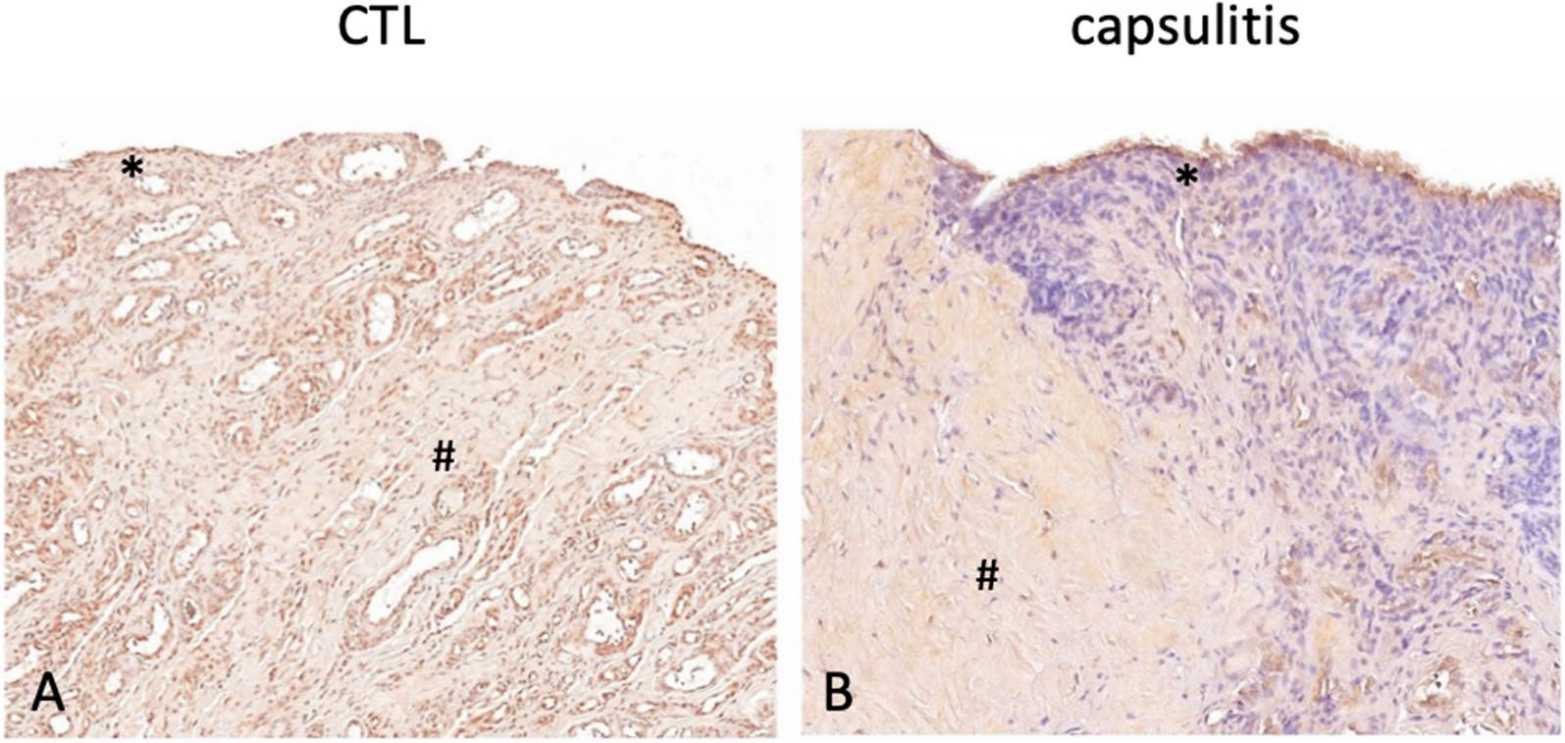
Immunohistochemical staining for lamin A/C in nuclei of fibroblasts (#) and synoviocytes (*) in controls (CTL, A) and in capsulitis samples (B). Nuclei were counterstained using hematoxylin dye. Original magnification: 20 ×

Immunohistochemical staining for lamin A/C revealed a generalized nuclear staining both in fibroblasts and in synoviocytes, with a lower expression in AC samples in comparison with controls, as shown in Fig. 1.

**Fig. 2:**
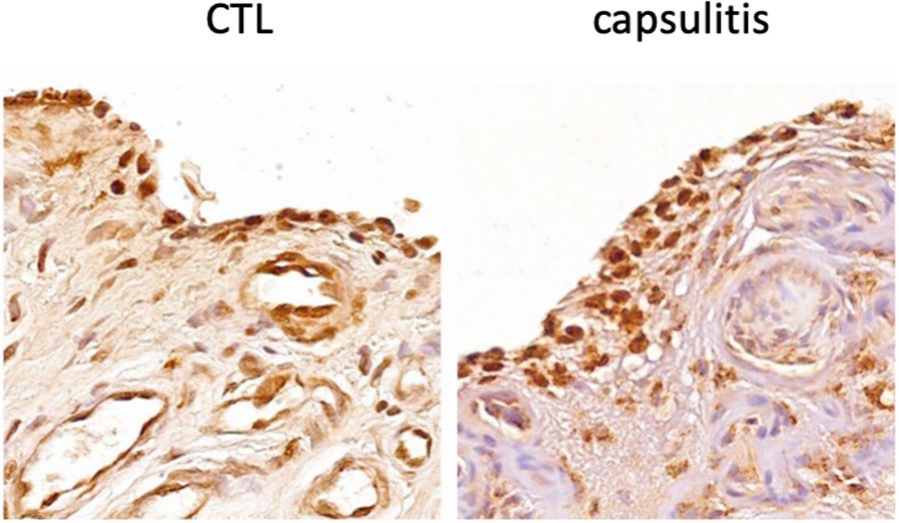
Lamin A/C expression in synoviocyte nuclei assessed in controls (CTL) and in adhesive capsulitis samples. Nuclei were counterstained by hematoxylin dye. Original magnification: 4 ×

**Fig. 3:**
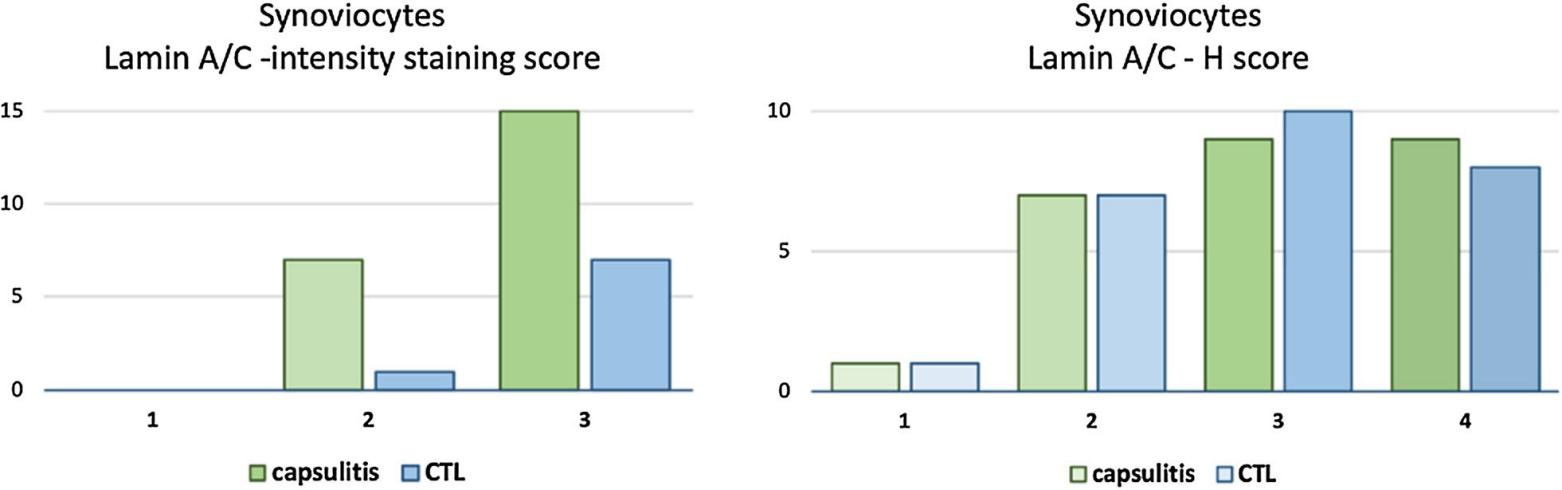
Distribution of lamin A/C immunohistochemical staining intensity and H-score in the synoviocytes. No differences were found between controls (CTL) and capsulitis samples

A deeper evaluation of lamin A/C expression demonstrated no differences in synoviocytes between cases and controls according to both lamin A/C intensity staining and H-score (p > 0.05) (Figs. 2, 3).

**Fig. 4:**
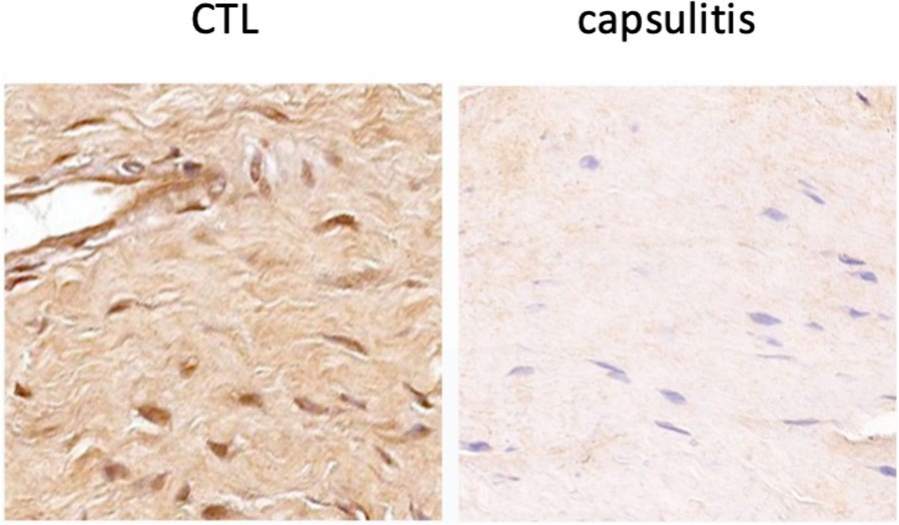
Lamin A/C expression in fibroblast nuclei in controls (CTL) and capsulitis samples. Nuclei are counterstained by hematoxylin dye. Magnification: 4 ×

**Fig. 5:**
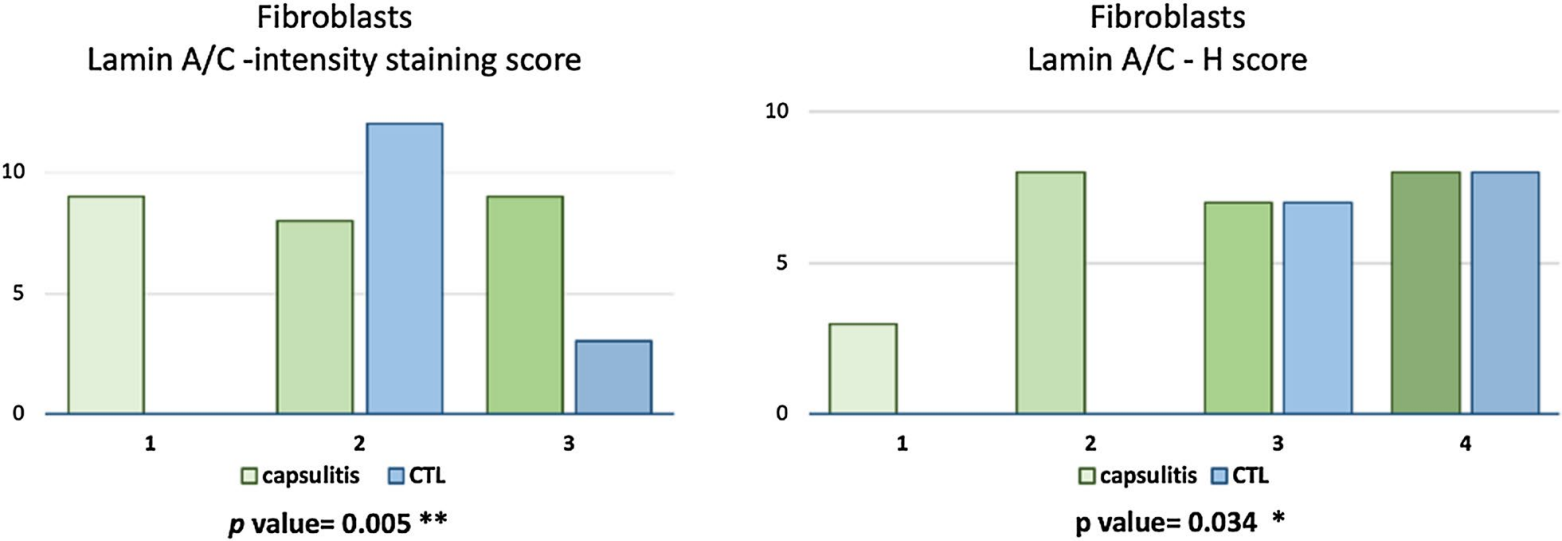
Distribution of lamin A/C immunohistochemical staining intensity and H-score in fibroblasts. A significant difference was found between controls (CTL) and capsulitis samples

As for fibroblasts, a panel of lamin A/C immunohistochemical staining is shown in Fig. 4. A significantly lower expression of lamin A/C was found in capsulitis samples in comparison with controls regarding both intensity of staining (*p*: 0.005) and H-score (*p*: 0.034) (Fig. 5).

**Fig. 6:**
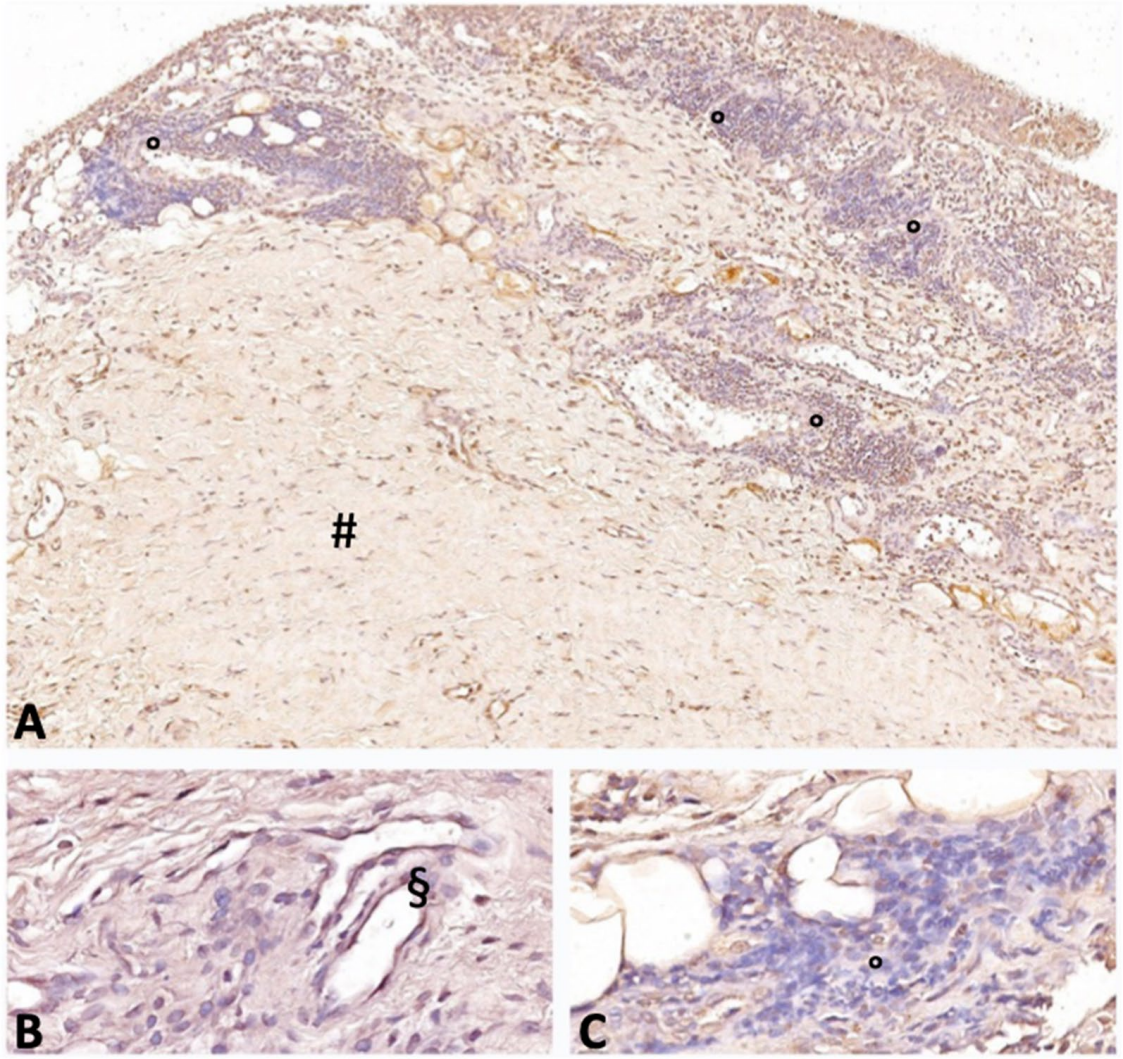
Immunohistochemical staining for lamin A/ in capsulitis samples in areas with (°) and without (#) acute inflammatory infiltrate. Nuclei are counterstained by hematoxylin dye (§). Magnification: 4 × in panel **A**, 20 × in panels **B**, **C**

**Fig. 7:**
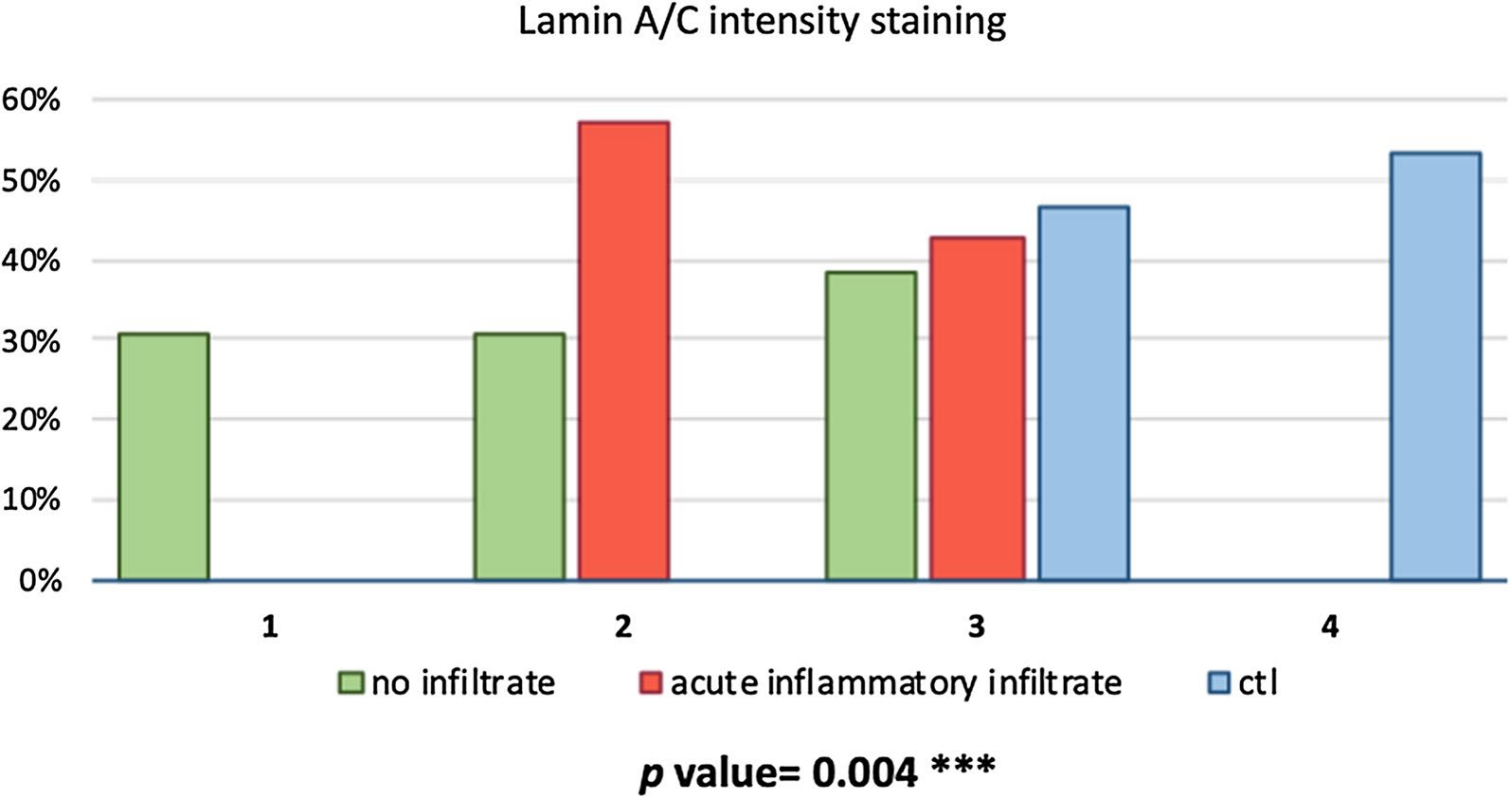
Distribution of lamin A/C immunohistochemical staining intensity in patients with and without acute inflammatory infiltrate. *CTL* controls

Considering only patients with AC, lamin A/C intensity staining was found to be significantly higher in samples where acute inflammatory infiltrate was detected (*p*: 0.004, Figs. 6, 7).

**Fig. 8:**
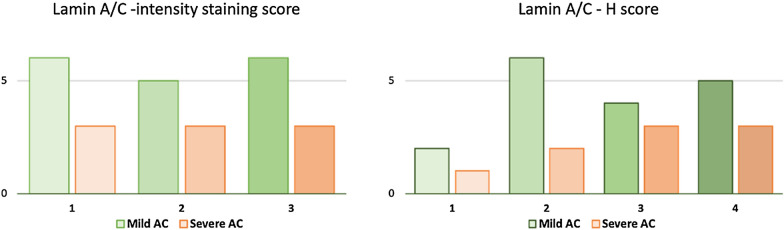
Distribution of lamin A/C immunohistochemical staining intensity and H-scores in patients with mild and severe adhesive capsulitis. *AC* adhesive capsulitis

Regarding the severity of adhesive capsulitis, 17 and 9 patients suffered mild and severe disease, respectively [mean forward flexion: group A = 144.3° (SD: 7.4°); group B = 79.6° (SD: 5.6°)]. Figure 8 shows the intensity of staining and the H-score of patients with mild and severe adhesive capsulitis. No differences were found between groups (*p*: > 0.05).

## Discussion

The present study is the first to search for the relationship between lamin A/C dysfunction and adhesive capsulitis. Immunohistochemical assessments of lamin A/C, such as its intensity and nuclear H-scores in fibroblasts and synoviocytes within rotator interval samples, obtained from patients with adhesive capsulitis were compared with controls. The results confirmed our hypothesis: a significant decreased expression of lamin A/C was found in fibroblasts of patients with adhesive capsulitis. Interestingly, no differences were found in synoviocytes between the groups. These findings confirm the recent evidence of Akbar et al. [18] on the importance of the fibroblast role in the etiopathogenesis of adhesive capsulitis as opposed to synoviocytes, which maintain cellular functions.

The altered lamin A/C expression in patients with adhesive capsulitis may be owing to:

- (1) The increased immunoreactivity to the advanced glycation end-products (AGE) documented in patients with AC. Hwang, et al. [19] immunohistochemically examined the relation of adhesive capsulitis to advanced glycation end-products and found a stronger immunoreactivity in tissue samples taken from the rotator interval of patients affected with adhesive capsulitis when compared with control groups. In their series both diabetic and non-diabetic patients affected with adhesive capsulitis expressed an increased immunoreactivity to the AGEs. Methylglyoxal (MGO)-derived AGE modifications were studied on vascular structures and theorized that some cellular components could be targeted for those modifications, among which are the intermediate filaments lamin A/C [20]. It is plausible that AGEs, acting on the capsular cells of the shoulder joint, affect its proteinaceous structures with a tendency to decrease lamin A/C expression which led to nuclear instability and AC onset in our group.
- (2) Modification of matrix metalloproteases (MMPs) in patients with adhesive capsulitis. In 1998, Hutchinson et al. [21] reported the results of a series of 12 patients with gastric carcinoma who received treatment with synthetic inhibitors of matrix metalloproteases; in a time range of 4 months, half of them developed adhesive capsulitis bilaterally. Soon after, in 2000, Bunker et al. [22] evaluated a variety of MMPs cytokines and growth factors in samples taken from the rotator interval of patients affected with adhesive capsulitis. It was demonstrated that the mRNA expression of MMP-2 in samples from patients with adhesive capsulitis was found more often than MMP-1 or MMP-3. Interestingly, from all patients affected with adhesive capsulitis, mRNAs for MMP-14, which has a significant role in MMP-2 activation, were not detected. When the peripheral blood values of matrix metalloproteases and tissue inhibitors of metalloproteases (TIMP) in patients with adhesive capsulitis were examined, MMP-1 level was significantly lower when compared with the control group whereas the TIMP levels were increased [23]. By inhibiting MMP-1, it was found that lamin A/C levels experienced faster degradation, suggesting that MMP-1 could play a role in the regulation of different factors involved in lamin A/C degradation [24]. Considering that MMP-1 is a factor commonly associated with adhesive capsulitis, it is possible that the relationship of MMP-1 to lamin A/C could contribute to the explanation as to why weaker expression of lamin A/C in patients with adhesive capsulitis were documented.
- Alternatively, other metalloproteases as MMP-2 and MMP-9 were overexpressed when examined by immunohistochemistry in capsules from patients with adhesive capsulitis [25]. An in vitro and in silico analysis on rat cardiomyocytes was performed and examined the proteolytic activity of MMP-2 on A- and B-type lamins, it was concluded that there is a susceptibility of lamin A/C to a proteolytic action caused by MMP-2, whereas no proteolytic changes by MMP-2 were documented in B-type lamins [26].
- (3) The relationship with telomere length. Telomeres are protein–DNA complexes that protect the ends of chromosomes from being detected as DNA breaks and from being processed [27]. Kalson et al. [28] studied the mean leukocyte telomere length (LTL) in a cohort study which checked for the correlation between joint fibrosis and telomeres. The authors concluded that in patients with adhesive capsulitis an altered telomere length was present and it could lead to severe tissue fibrosis. Interestingly, it was established that the loss of lamin A/C leads to telomere shortening and inevitably to the loss of nuclear stability [29]. Future studies should investigate the length and function of telomeres and their relation to lamin A/C in fibroblasts and synoviocytes in patients with adhesive capsulitis specifically within the rotator interval.

In our group of patients with AC, significantly higher expression of lamin A/C was correlated with the presence of acute inflammatory infiltrate in samples obtained from the rotator interval. This finding demonstrated that the phase of the disease plays a fundamental role in maintaining the functionality of the cell nucleus in patients affected by adhesive capsulitis. In fact, inflammatory alterations with synovial hyperplasia, fibroblastic proliferation and increase in the quantity of fibroblasts can occur early in the pathogenesis of adhesive capsulitis [30]. A progressive fade away of the inflammatory signs can take place in the later stages of the disease as the patient already suffers from pain and shoulder range of motion limitation [31]. When examining the environment of the tissue at this late of a stage, large quantities of fibroblasts are found to be differentiated into myofibroblasts and located within the extracellular matrix (ECM) [32].

On the contrary, the clinical severity of adhesive capsulitis did not correlate with the functionality of the cell nucleus in our samples; in fact, no differences were found regarding lamin A/C expression between patients with less and more severe adhesive capsulitis.

Our study also demonstrated that adhesive capsulitis does not completely compromise cell stability, contrary to what has been recently documented in tenocytes of patients with massive rotator cuff tear [9]. In fact, mainly diminished lamin A/C expression was detected, while no samples demonstrated absence of lamin A/C.

Our findings stress the importance of early and aggressive treatment in patients with adhesive capsulitis starting from the initial and less severe stages of the disease (freezing and early frozen). In patients with conservative treatment failure, surgery should not be postponed as the progression of the disease leads to the loss of lamin A/C function on the nuclear activity.

Altered lamin A/C expression has been described in other orthopaedic pathological conditions, including cancer, such as paediatric osteosarcoma, lamin A/C scales with tumour aggressiveness [33] and the restoration of lamin A/C expression in highly aggressive osteosarcoma cells reduced cancer cell migration [34] and aggressiveness [35]. In view of this, our study shed light on the relevance of lamins and the mechanotransduction process in orthopaedic diseases, both in adult and paediatric patients.

The present study has limitations that need to be addressed: the relatively low number of studied patients and the low number of controls. However, the evaluation of lamin A/C immunohistochemical staining is a novel and expensive methodology.

## Conclusions

Our study demonstrated that the activity of lamin A/C in maintaining nuclear structural integrity and cell viability is decreased in patients with adhesive capsulitis. The phase of the disease is the key factor for cell functionality; in fact, cellular functions are maintained in the early stages of the disease. Conversely, the clinical severity of adhesive capsulitis plays a marginal role.

## Data Availability

Available upon request.
